# Differentiated Weed-Suppressive Ability of Modern and Old Durum Wheat Cultivars after Long-Term Cultivation under Semi-Arid Climate

**DOI:** 10.3390/plants11233368

**Published:** 2022-12-04

**Authors:** Aurelio Scavo, Alessia Restuccia, Mario Bannò, Giovanni Mauromicale

**Affiliations:** Department of Agriculture, Food and Environment (Di3A), University of Catania, 95123 Catania, Italy

**Keywords:** *Triticum durum*, weed management, soil seedbank, species diversity, weed communities, old landraces, multivariate statistics

## Abstract

Durum wheat (*Triticum turgidum* spp. *durum*) is one of the most important grain crops cultivated across the Mediterranean Basin, where a strong return to local landraces cultivation is occurring to meet the market demand for high-quality food and low-input cropping systems. A characterisation of the long-term effect (10 years) of durum wheat landraces and modern cultivars on the potential and real weed flora is still lacking. Hence, a multilocation trial over 10 farms in Central-Eastern Sicily was carried out to investigate the repeated cultivation of several old landraces (OLD) and modern cultivars (MOD) on the abundance and diversity of weed flora. Overall, OLD was associated with a 47% reduction of the soil seedbank size and to −64% of the aboveground weed biomass compared to MOD. In addition, diversity indices pointed out a high similarity between MOD and OLD farm groups for the soil seedbank, while a lower diversity was found in OLD for aboveground weed communities. From the principal component analysis emerged that the species compositions of MOD and OLD were quite separated for both soil seedbank and real flora, with the latter showing few specific associations with major weeds. These findings demonstrated the indirect effect of durum wheat landraces in sustainably reducing weed pressure without the adoption of chemical weed control.

## 1. Introduction

Durum wheat (*Triticum turgidum* subsp. *durum* (Desf.) Husn., 2*n* = 4*x* = 28, AABB), although grown on just 8–10% of the global land surface, is one of the most important cereal crops in semi-arid zones, especially in the Mediterranean Basin, where more than 80% of the total European-harvested production take place [1]. In Europe, Italy is the first country of economic importance with 3.8 × 10^6^ Mg of harvested production obtained from 1.2 × 10^6^ ha [2], primarily concentrated in the southern regions. Mediterranean durum wheat germplasm is characterised by the largest biodiversity, as demonstrated by the high number of local landraces adapted to numerous pedoclimatic conditions [3]. However, many of these old landraces are no longer cropped or are underutilised due to the spread of modern genetically improved and high-yielding cultivars, thus causing a serious genetic erosion [4,5].

Aside from major abiotic constrains such as drought, nitrogen supply, high temperatures and soil properties, weeds are the most important biotic threat reducing the yields and quality characteristics of durum wheat [6,7], especially in the Mediterranean Basin. To control weeds, over the last decades, durum wheat has been subjected to a considerable chemical weed control that caused a number of negative effects, mainly the development of highly resistant weed populations and the persistence of herbicides in the environment and in the food chain. Considering the adverse effects determined by the irrational chemical weed control, on the one hand, and the raising of organic cropping systems where synthetic herbicides are banned, on the other hand, the exploitation of eco-friendly weed management practices in durum wheat agroecosystems has an outstanding relevance [8]. In recent years, several sustainable weed management practices involving the manipulation of allelopathy, such as the use of plant water extracts [9], intercropping [10] and mulching [11], have been proposed in durum wheat [12]. Within the integrated weed management (IWM) systems, a key role is played by prevention, including those strategies or agronomic choices aimed at preventing weed adaptation, and it is based on the reduction of the soil seedbank and the improvement of the crop competitiveness against weeds [8]. The former is the most important and challenging aspect, given that the soil seedbank is the primary source of new infestations and that the real weed flora derives almost exclusively from the potential weed population communities [13]. The main goal is getting its control below 20 million weed seeds ha^−1^ in order to simplify and reduce the direct weed control methods. The second strategy, which is closely connected to the soil seedbank control, can be addressed by choosing weed-competitive cultivars with high root development, early vigour, faster seedling emergence, high growth rates, wide leaf areas and an allelopathic ability [14]. In this regard, several findings suggest a higher weed-suppressive ability of old durum wheat landraces than modern cultivars due to a combined competitive–allelopathic effect [15,16]. Fields of old durum wheat landraces, in fact, generally show lower weed densities than modern cultivars, according to our experience. This aspect, together with the increasingly importance of low-input agricultural systems (especially in the European Union) and a greater market demand for high-quality food, is determining a reawakened interest in durum wheat local landraces. They are particularly appreciated by the market by virtue of their high-quality flours, especially for the production of pasta, pizza and bread.

Based on these considerations, the present research started from the hypothesis that durum wheat landraces may have a higher weed-suppressive ability than modern cultivars and that the repeated cultivation of local landraces may reduce the soil seedbank (potential weed flora) and the real weed flora pressure. A scientific verification directly on a field scale has never been done. Hence, the goals were to evaluate the effect derived from the long-term rotation, including some old durum wheat landraces, compared to modern cultivars, on the abundance and diversity of potential and real weed flora in Central-Eastern Sicily, a semi-arid environment representing an important production centre of durum wheat.

## 2. Results

### 2.1. Potential Weed Flora (Soil Seedbank)

#### 2.1.1. Weed Abundance

**Figure 1:**
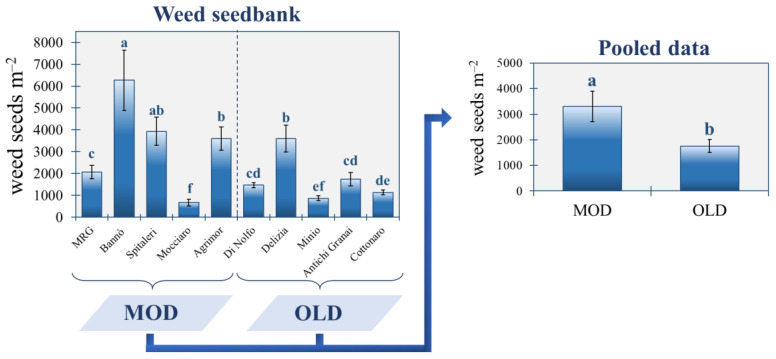
Size of the weed soil seedbank (0–15 cm) across the 10 farms under study. Bars are the standard deviation (*n* = 3). Different letters indicate statistical significance by applying one-way analysis of variance with Tukey’s HSD test at *p* < 0.05. MOD: wheat modern varieties group; OLD: wheat old landraces group.

Pooling over the five farms belonging to the MOD and OLD groups (Figure 1), it is clearly visible that the repeated cultivation of old durum wheat landraces was associated with a 46.8% reduced seedbank size compared to modern cultivars in the studied area (1760.0 vs. 3306.7 seeds m^−2^). From the analysis of variance (ANOVA), it emerged that the soil seedbank varied significantly across the ten farms under study (Figure 1). In detail, the highest seedbank size was found at the Bannò Farm (6266.7 seeds m^−2^), where the cv. Anco Marzio is cultivated from many years in rotation with vetch, followed by the Spitaleri Farm (3933.3 seeds m^−2^), which sows an Iride–Simeto–Core mix in rotation with vetch and fava beans. The lowest seedbank size was detected at the Mocciaro Farm (666.7 seeds m^−2^), despite it sowing modern cv. Core alternately with a fodder mix composed of vetch, clover, Sulla, ryegrass and oat, followed by Minio (866.7 seeds m^−2^), which sows the old cv. Perciasacchi. Delizia, which also cultivates the cv. Perciasacchi, and which is the only farm adopting the stale seedbed, showed the highest seedbank size (3600.0 seeds m^−2^) within the OLD group.

#### 2.1.2. Weed Diversity

Throughout the ten farms, the total 0–15 cm soil seedbank consisted of 13 weed species or genera belonging to 11 botanical families (Table 1). All detected taxa were annual therophytes, excluding the biennial hemicryptophyte *Stellaria media* (L.) Vill. The soil seedbank was dominated by six major weeds (i.e., with a RD ≥ 5%): in decreasing order, *Euphorbia helioscopia* L., *Anagallis arvensis* L., *Helminthotheca echioides* (L.) Holub, *S. media*, *Sinapis arvensis* L. and *Fallopia convolvulus* (L.) Á. Löve. Their sum accounted for 86% of the total weed seedbank density. The ANOVA of the species richness and RAIs did not show significant differences among the MOD and OLD for each species (data not shown). However, Table 1 highlights some interesting findings. About the major weeds, *E. helioscopia* and *S. arvensis* were more abundant at the Delizia Farm (0.54 and 0.22, respectively), *A. arvensis* at Antichi granai (0.72), *E. echoiides* at MRG (0.38), *S. media* at Di Nolfo (0.45) and *F. convolvulus* at Minio (0.28). Moreover, the weeds *Glebionis coronaria* (L.) Cass. ex Spach, *Portulaca oleracea* L. and *Veronica* sp. were detected only in MOD, whereas *Galium aparine* L. and *S. media* were exclusive of OLD. Interestingly, *A. arvensis, E. helioscopia*, *F. convolvulus* and *Fumaria* sp. had higher mean RAIs in the MOD farm group, while the RAIs of *Amaranthus retroflexus* L., *E. echoiides* and *S. arvensis* were higher in farms belonging to the OLD group.

Taking into account the α-diversity, no significant differences were observed for the Margalef’s (D_MG_), Shannon–Wiener (H) and Pielou’s (J) indices between the MOD and OLD (Table 2). Within the MOD, Agrimor, cultivating cv. Core in rotation with vetch, showed the highest D_MG_ (2.08) and H (1.48) values, indicating a higher biodiversity compared to the other MOD farms. On the contrary, Mocciaro, which was the farm with the lowest seedbank size, had the highest J value (0.94), indicating the presence of a few dominant species, namely *A. arvensis*, *E. helioscopia* and *P. oleracea* (Table 1). Within OLD, similarly to the Mocciaro Farm, Minio showed the highest J (0.91), while Antichi granai had the lowest, α-diversity indices. Concerning β-diversity (Table 2), a high similarity between the MOD and OLD groups was found both in terms of presence/absence (Sørensen’s, 76.2%) and abundance (Steinahus’s, 54.7%).

### 2.2. Real Weed Flora

#### 2.2.1. Weed Abundance

**Figure 2:**
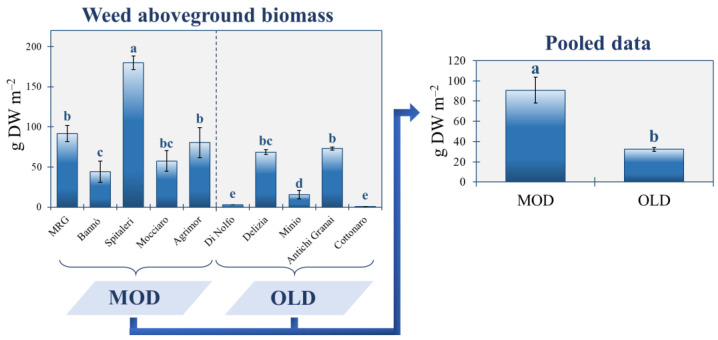
Aboveground biomass (g DW m^−2^) of the real weed flora across the 10 farms under study. Bars are the standard deviation (*n* = 3). Different letters indicate statistical significance by applying one-way analysis of variance with Tukey’s HSD test at *p* ≤ 0.05. MOD: wheat modern varieties group; OLD: wheat old landraces group.

Averaged over the MOD and OLD (Figure 2), emerged durum wheat landraces may have determined a 64.4% reduction of the real weed flora abundance in the studied area with respect to modern cultivars (2.1 vs. 12.7 g DW m^−2^). Similar to the soil seedbank, ANOVA showed that the real weed flora abundance was significantly different for the ten farms (Figure 2). The Spitaleri Farm, in particular, had the highest weed aboveground biomass (180.1 g DW m^−2^), followed by MRG (91.6 g DW m^−2^) and Agrimor (80.4 g DW m^−2^). Within OLD, the highest weed aboveground biomass was found at Antichi granai (73.0 g DW m^−2^), which is the only farm performing fertilisation, and Delizia (68.6 g DW m^−2^), the farm with the highest seedbank size. At Cottonaro, which carries out a long-term rotation durum wheat cv. Senatore Cappelli with a leguminous mix (vetch, clover and Sulla) and which is the only OLD farm performing chemical weed control, no emerged weeds were detected.

#### 2.2.2. Weed Diversity

Nineteen weed species or genera were recorded throughout the study in the real weed flora, of which 74% were annuals, 16% perennials and just one biennial hemicryptophyte, namely *S. media* (Table 3). Among the 19 detected taxa, 39% belong to Asteraceae, 23% to Poaceae and 15% to Brassicaceae. Seven weeds or genera had a RD ≥ 5% and thus dominated the real weed flora: *Avena fatua* L., *S. media*, *G. aparine*, *G. coronaria*, *Lolium* sp., *Centaurea* sp. and *Phalaris paradoxa* L., which, altogether, accounted for 71.2% of the total density. As observed for the soil seedbank, although the ANOVA of the species richness and RAI was not significant, the following statements could be highlighted: *A. fatua* and *Lolium* sp. were more abundant at the Mocciaro Farm (0.54 and 0.31, respectively), *S. media* and *G. aparine* at Di Nolfo (0.27 and 0.54, respectively), *G. coronaria* at Spitaleri (0.27), *Centaurea* sp. at MRG (0.18) and *P. paradoxa* at Agrimor (0.23). Furthermore, *Artemisia vulgaris* L., *Inula helenium* L., *Papaver rhoeas* L., *Polygonum aviculare* L. and *S. arvensis* were recorded only at the MOD farms, whereas *Convolvulus arvensis* L., *Diplotaxis erucoides* (L.) DC., *E. helioscopia*, *Erodium cicutarium* (L.) L’Hér. and *G. aparine* only at the OLD. From Table 3, it is also possible to see that *Centaurea* sp., *G. coronaria*, *P. paradoxa* and *Sonchus* sp. had a higher RAI in MOD, while *D. carota*, *Lolium* sp. and *S. media* showed a higher RAI in OLD.

In contrast with the soil seedbank, for the real weed flora, the α-diversity was significantly higher in the MOD farm group than the OLD for the D_MG_, H and J (Table 4). In detail, MRG had the highest α-diversity (D_MG_ = 3.6; H = 1.9; J = 0.8) across the MOD farm group, while Di Nolfo showed the lowest values (D_MG_ = 1.2; H = 0.8; J = 0.7) across the OLD farms. Despite significant α-diversity differences, the MOD and OLD showed a medium-high qualitative β-diversity (Sørensen’s = 64.3%) but a Steinahus’s coefficient < 50%.

### 2.3. Species Composition of Potential and Real Weed Flora

**Figure 3:**
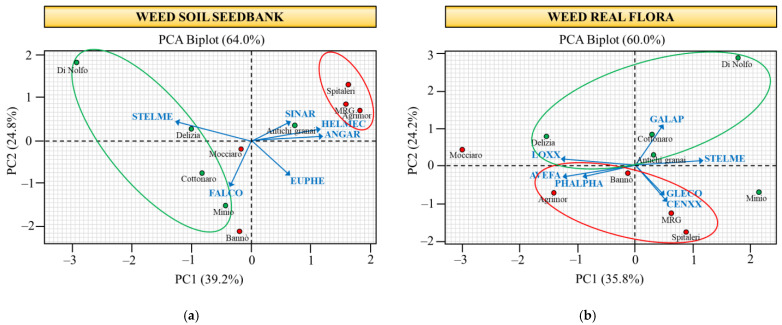
Principal components analysis ordination biplot from the correlation matrix with the 6 major weeds for the soil seedbank (**a**) and with the 7 major weeds for the real weed flora (**b**) across the 10 farms under study. Farms belonging to the MOD group are labelled green, while farms from the ANT group are shown with red circles. Arrows highlight the discrimination of weeds along the principal components. Groups: MOD: wheat modern varieties; OLD: wheat old landraces. Weeds: ANGAR (*Anagallis arvensis*), AVEFA (*Avena fatua*), CENXX (*Centaurea* sp.), EUPHE (*Euphorbia helioscopia*), FALCO (*Fallopia convolvulus*), GALAP (*Galium aparine*); GLECO (*Glebionis coronaria*), HELMEC (*Helminthotheca echioides*), LOXX (*Lolium* sp.), PHALPHA (*Phalaris paradoxa*), SINAR (*Sinapis arvensis*) and STEMLE (*Stellaria media*).

The associations between major weeds and farms were analysed by PCA on the correlation matrix of standardised weed densities. The eigen analysis showed that, for both potential and real weed flora, the first three PCs gave eigenvalues greater than one and accounted for most of the variance (Table 5). Interrelationships among major weeds and farms were observed graphically through ordination biplots constructed with the first two components explaining the maximum variance. For the soil seedbank, *A. arvensis*, *H. echioides* and *S. media* captured 67.4% of the variance in PC1, and *E. helioscopia* and *F. convolvulus* added a 59.8% variance in PC2, while *S. arvensis* had the highest weight on PC3. In addition, PC1 was positively correlated to *A. arvensis*, *E. helioscopia*, *H. echioides* and *S. arvensis*, thus positioning them on the right side of the biplot (Figure 3), while a negative correlation (left side) was found with *F. convolvulus* and *S. media*. PC2 correlated positively (top of the biplot) with *A. arvensis*, *H. echioides*, *S. arvensis* and *S. media* and negatively (bottom) with *E. helioscopia* and *F. convolvulus*. For the real weed flora, *A. fatua*, *Lolium* sp. and *S. media* accounted for 59.7% of the PC1 variance, *Centaurea* sp. and *G. aparine* for 54.1% in PC2 and *G. coronaria* and *P. paradoxa* for 39.5% in PC3 (Table 5). Moreover, the weeds *Centaurea* sp., *G. aparine*, *G. coronaria* and *S. media* showed a positive correlation with PC1, whereas *A. fatua*, *Lolium* sp. and *P. paradoxa* correlated negatively. Except for *G. aparine*, *Lolium* sp. and *S. media*, all weeds correlated negatively with PC2 (bottom of the biplot). The ordination biplots show that the farms discriminated mainly along PC1 for the soil seedbank and along PC2 for the real weed flora (Figure 3). In particular, except for Antichi granai, the OLD farms were positioned on the left side of the soil seedbank biplot; about real flora, all the OLD farms, excluding Minio, were positioned on the top of the biplot. Therefore, the MOD and OLD were quite separated for both soil seedbank and real flora, with OLD farms that showed few specific associations with the major weeds. Di Nolfo, in particular, was not associated with any species.

## 3. Discussion

In this research, the weed-suppressive ability of the modern and old durum wheat cultivars was evaluated on both the soil seedbank and real weed flora under a semi-arid climate, namely Central-Eastern Sicily, an important Mediterranean centre production of such a crop. Following Travlos et al. [17] and Nkoa et al. [18], weed abundance and diversity were considered. Though the obtained results were farm-specific, pooling over farms emerged clearly that old landraces, compared to modern cultivars, were associated with a high decrease of soil seedbank, seed emergence and weed growth, as indicated by the lower aboveground biomass weight, despite four of the five farms cultivating old landraces not performing chemical weed control. Nevertheless, four of the five farms belonging to OLD group showed a seedbank size < 20 million seeds ha^−1^, which is an important goal to reduce the direct weed control methods within IWM strategies. This significantly higher weed-suppressive ability of old landraces could be attributed to their greater competitive traits and allelopathic properties, as well as to the differences in the management practices performed. The crop competitive ability is conferred by a number of morphophysiological traits, such as fast seedling emergence, early vigour, high growth rates and root development, plant height, leaf area index, etc. [8,9,10,11,12,13,14]. Mwendwa et al. [19], for instance, reported a higher capacity in suppressing weed establishment by those bread wheat (*T. aestivum*) cultivars showing early vigour and early canopy closure, high biomass production and height. Lemerle et al. [15], screening several Australian wheat genotypes for their competitiveness against weeds, found that durum wheats were less competitive than *T. aestivum* and that old landraces suppressed the weeds more than all modern cultivars. Giambalvo et al. [20], after comparing one durum wheat landrace and two modern cultivars for their nitrogen use efficiency under induced interspecific competition, reported a higher competitive ability of the landrace Russello, likely due to its capacity in reducing the N availability to a competitor, a factor that increased with the increasing plant stature. About wheat allelopathy, the literature refers that the concentration of wheat allelochemicals, mainly belonging to benzoxazinoids, phenolic acids and short-chain fatty acids [20,21], varies considerably based on the cultivar choice [22]. In this regard, recently, Scavo et al. [16] found that three durum wheat old landraces (Timilia, Russello and Perciasacchi) were able to reduce seed germination and increase the mean germination time of the weeds *P. oleracea* and *S. media* more than the modern cultivar Mongibello. The authors supposed that the improved phytotoxicity of old landraces might be caused by their higher total polyphenol and total flavonoid contents. Lo Bianco et al. [5] indicated a specific and genotype-dependent pattern of phenolics concentration among ten Sicilian durum wheat landraces and three genetically improved cultivars, with coumarin, vanillic acid, luteolin and apigenin conjugates that were more abundant in local landraces. These phenols are recognised as well-known allelochemicals against several weeds [23]. In addition, Di Loreto et al. [24] reported a twofold greater content of vanillin, *p*-coumaric acid and 4-hydroxybenzaldehyde, as well as a 1.6-times greater amount of ferulic acid and syringaldehyde in old landraces than in modern cultivars. Here, it is likely that, in addition to their highly competitive traits, the repeated cultivation of durum wheat old landraces caused a build-up of allelochemicals into the rhizosphere through root exudation and plant residue decomposition. To reinforce this hypothesis, Belz and Hurle [25], after screening 146 cultivars of four Triticeae species, including durum wheat, demonstrated a high cultivar dependence in benzoxazinoids exudation, with hexaploid species that accumulate preferentially DIMBOA and only low levels of DIBOA, while the tetraploid *T. durum* accumulates substantial levels of both glucosides. Once in the rhizosphere, these allelochemicals interact with the complex of soil physical, chemical and biological characteristics that affect their bioavailability and phytotoxic level [26].

About species diversity, in this study, the potential and the real weed flora showed some common aspects, taking into account that emerged weeds are derived, to a large extent, from the soil seedbank. Both types of flora were largely composed of therophytes and annual weed species, as common under semi-arid climates [27], and were dominated by the few species present at high density. Moreover, in both cases, no significant differences were observed in terms of the species richness. Indeed, the diversity indices pointed out a high similarity between the MOD and OLD farm groups for the soil seedbank in terms of both the α- and β-diversity. On the contrary, in the real weed flora, the α-diversity was significantly higher in the MOD than the OLD farm group, and the two groups showed a quantitative β-diversity below 50%, the limit below which a dissimilarity can be interpreted. The values obtained here are in line with Hyvonen et al. [28], who registered values ranging from 50% to 80% in weed communities of cereal crops under a temperate climate. To better visualise the species compositions among the MOD and OLD farm groups, a PCA was carried out on the major weeds of both potential and real weed flora. The biplots showed that the magnitude of changes in the weed community composition varied between the potential and real weed flora, thus indicating a low correspondence between the below and aboveground weed communities, in accordance with Cardina and Sparrow [29]. The values of the seedbank size and aboveground biomass, in fact, did not always correspond with each other. Davis et al. [30] also reported little predictive value between the weed seedbanks and weed biomass within a long-term corn–soybean–wheat crop sequence under conventional and no tillage systems. In contrast with our findings, Ghersa and Ghersa-Martinez [31] indicated a strong predictive capacity of potential flora for aboveground weed communities in no tillage systems, because the shallow depth placement of seeds leads to greater proportional recruitment. Therefore, it is likely that the low correspondence between the soil seedbank and aboveground weed communities detected here is due to tillage systems performed in the studied area. In both the soil seedbank and real weed flora, however, the MOD and OLD farm groups were quite separated, suggesting different shifts and patters of weed communities between the modern cultivars and old durum wheat landraces. Moreover, the OLD farm group showed a few specific associations with major weeds, meaning that the local landraces were associated, to a lesser extent, with the major weeds than the modern cultivars.

## 4. Materials and Methods

### 4.1. Description of Survey Area

**Figure 4:**
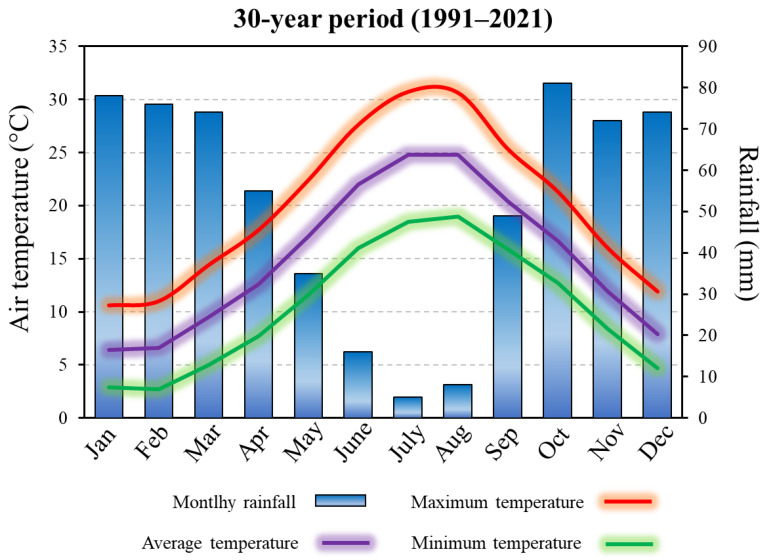
Long-term period of total monthly rainfall and monthly air temperatures (maximum, average and minimum) in the survey area (Central-Eastern Sicily, South Italy).

The present research was performed across 10 wheat farms located in Central-Eastern Sicily, an area devoted to cereal cultivation with a long tradition of durum wheat production. The climate of the zone is semi-arid Mediterranean, characterised by dry, long summers and mild, wet winters. The average annual rainfall in the last 30 years was 623 mm, mainly distributed over the autumn–winter period (Figure 4). Mean monthly air temperature is 15.1 °C, with July and January as the months with, respectively, the highest (24.8 °C) and the lowest (6.4 °C) temperatures (Figure 4). According to the USDA classification [32], the soils are Regosoils (Typic Xerorthensis or Xerochrepts) and Alluvial (Typic Vertic Xerofluvents), with moderately clayey texture.

### 4.2. Agronomic Management

In order to study the long-term effects derived from the repeated cultivation of some old landraces and modern durum wheat genotypes on weed flora, 10 cereal farms were selected for their long-term cultivation of durum wheats. Half of them grow modern durum wheat varieties, and the other half cultivates old landraces. Table 6 and Table 7 show the geographical coordinates and the agronomic management of the ten farms. The modern durum wheat cultivars under study, characterised by early or early-medium maturity, were Antalis; Anco Marzio; Core and a mix composed of Iride, Simeto and Core. Old durum wheat landrace, showing a medium-late cycle, were Perciasacchi, Timilia and Senatore Cappelli. In the studied zone, they are usually sown in late autumn, with a seeding rate ranging from 160 to 300 seeds m^−2^, and harvested in late spring or at the beginning of summertime [33]. All farms cultivating modern cultivars controlled the weeds chemically and applied a mineral fertilisation. On the contrary, among the farms cultivating old landraces, Antichi granai was the only one that fertilised the crop and Cottonaro the only farm performing weed chemical control. In addition, only the Delizia Farm carried out the stale seedbed. Long-term crop sequences of the ten farms are shown in Table 8.

### 4.3. Weed Flora Analysis

Both potential (soil seedbank) and real weed flora (aboveground species) were analysed by considering the abundance and diversity. Prior to sampling, the study sites were monitored with a field scouting to visualise weed distribution and locate the sampling units, excluding the borders of each plot and the nonrepresentative areas. Due to the variation between and within the study sites, a stratified random sampling was adopted [18], which consisted of dividing each sampling zone into homogeneous strata. In detail, a 1000 m^2^ area was selected in each farm—within which, three sampling zones were located. Soil and aboveground weed samples were collected on June 2nd and 3rd 2022, just before wheat harvest.

#### 4.3.1. Soil Seedbank (Potential Weed Flora)

Soil samples were taken with a core sampler 10–15 cm deep along the diagonals of the central part of each sampling zone [34]. A sample was obtained by pooling 5 randomly distributed subsamples, each 0.75 dm^3^, for a total of 150 soil cores (10 farms × 3 replicates × 5 subsamples) collected. Following Scavo et al. [13], the soil samples were freed from inert fraction (stones, pebbles and dead debris), and the seeds were extracted from the soil by using a metal tube (Karcher, K 3500 model, Winnenden, Germany) equipped with a removable cap consisting of a 250 μm steel mesh. For high clayey soils, a pre-treatment with 5 g of sodium hexametaphosphate solution for 20 min was necessary to disperse the colloid matrix. The count and identification of weeds was performed with a MS5 Leica stereomicroscope (Leica Microsystems, Wetzlar, Germany) in Petri dishes after 24 h of air-drying. The seedbank size was calculated as the number of seeds per square metre of surface area for each plot.

#### 4.3.2. Aboveground Species (Real Weed Flora)

The analysis of the real weed flora was carried out over three randomly placed quadrats (each of 1 m^2^) per sampling zone [27]. For the total aboveground weed biomass, weeds were clipped at the soil surface, dried to constant mass and weighed (pooled weight at the quadrat level was considered). In each quadrat, weeds were sorted by species or genera, together with the number of individual plants per species.

#### 4.3.3. Weed Abundance

Aside from the seedbank size for potential flora and aboveground biomass for real flora, weed species abundance was calculated under the relative density (RD), relative frequency (RF) and relative abundance index (RAI), in accordance with Scavo et al. [34]:

(1) $$\mathrm{RD}\;\left(\%\right)=\left(\frac{\sum Y_{i}}{S}\right)\times 100$$

(2) $$\mathrm{RF}\;\left(\%\right)=\left(\frac{F_{i}}{\sum F}\right)\times 100$$

(3) $$\mathrm{RAI}=\frac{\mathrm{RD}+\mathrm{RF}}{2}$$

where: ∑Y_i_ = sum of the number of individuals or seeds for a weed species, S = species richness within the plot, F_i_ = number of sampling units in which the species *i* occurred and ∑F = sum of the absolute frequencies of all species. The RAI is a valuable parameter in characterising weed communities, since it takes into account both the weed density and evenness, thus overcoming the problems caused by a nonuniform weed distribution [35].

#### 4.3.4. Floristic Composition and Species Diversity of Weed Communities

After seed or individual plant identification, the floristic composition was assessed based on Conti et al. [36], grouping weed species or genera by botanical family, life cycle and biological group. Species diversity was explored as richness and evenness. The former is the total number of weed species present in a community, and the latter provides information about the abundance of each species in a community [17]. Considering the variability across the study sites, the species diversity was estimated within the community (α-diversity) and between communities (β-diversity). Margalef’s (D_MG_), Shannon–Wiener (H) and Pielou’s (J) indices were computed for the α-diversity:

(4) $$D_{\mathrm{MG}}=\left(S-1\right)/\ln\left(N\right)$$

(5) $$H=-\sum p_{i}\ln p_{i}$$

(6) J = H/lnS

where N = total number of seeds or individuals of all species in the community, and *p_i_* = proportional abundance of the *i*th species. Margalef’s diversity index is a rapid method to measure the gross species diversity only based on richness, but it is very sensitive to the sample size. In contrast, J measures only the evenness, and H includes both species richness and evenness.

For the β-diversity, three common diversity indices were chosen, i.e., Whittaker’s statistics (W), Sørensen’s (S_S_) and Steinahus’s (S_A_) coefficients, in accordance with Ramírez et al. [37] and Restuccia et al. [27]:

(7) $$W=\frac{\Upsilon }{S}$$

(8) $$S_{S}=\left[\frac{2J}{\left(a+b\right)}\right]\times 100$$

(9) $$S_{A}=\left(\frac{2W}{A+B}\right)\times 100$$

where *Υ* = total number of all species in the entire study area, J = number of common species to each community; a + b = sum of the total number of species in each community, W = sum of the lower of the two abundances of each species in the community, A = total number of individuals in population A and B = total number of individuals in population B. Whittaker’s statistics measures the rate of species turnover, and S_S_ measures the binary similarity in terms of presence/absence, while S_A_ accounts for the differences in abundance.

### 4.4. Statistical Analysis

All data were subjected to ANOVA by applying a generalised linear model (GLM) with the protected Tukey’s HSD means separation test at α = 0.05. Prior to ANOVA, the normality and homogeneity of variance were respectively assessed with the Shapiro–Wilk’s and Bartlett’s tests. To meet the ANOVA assumptions, the seedbank and aboveground biomass data were log_(x + 1)_-transformed, the RAI data needed an arcsine–square root transformation and the H and J data were respectively square root- and logit-transformed, while the species richness showed a homogeneous variance distribution [34]. For an easier interpretation of the results, data from the five farms cultivating modern cultivars and from the other five farms cultivating old landraces were respectively pooled and presented into two groups: MOD and OLD. Multivariate statistics was used to analyse the weed community composition. Following Restuccia et al. [27], a principal component analysis (PCA) on the correlation matrix of the standardised major weeds (those with RD ≥ 5%) was performed on both the potential and real weed flora. The results were graphically presented on “distance” biplots derived from the first two principal components explaining the maximum variance [38]. Biplots allowed visualising the relations between the variables (major weeds) and wheat genotypes. Minitab^®^ version 16 (Minitab Inc., State College, PA, USA) statistical software was employed for all analyses.

## 5. Conclusions

The results obtained here, as a whole, demonstrate that the old landraces of durum wheat may possess a stronger weed-suppressive ability than modern cultivars in terms of seedbank and aboveground biomass reduction. Within the studied area, landraces were also able to reduce the aboveground weed species diversity and to cause shifts in weed populations. The importance of these findings is even greater if considering that four of the five farms cultivating old landraces did not perform chemical weed control. Therefore, this research provided the scientific basis for the increased interest that consumers, government policies and scientists have moved toward durum wheat landraces by virtue of their sustainable cultivation, high product quality and remuneration. On the other hand, although a multilocation trial involving ten different farms was carried out, our study considered just one growing season. Furthermore, it should be considered that the high heterogeneity in terms of the management practices across the farms under study may have affected the obtained results. In future steps, we aim to perform an economic analysis of these findings, as we noted that old landraces can be grown with lower inputs, and their products can be sold at a higher price than modern cultivars.

## Figures and Tables

**Table 1:** Mean relative abundance values and mean relative densities (RD) of weed species in the total seedbank (0–15 cm) across the 10 farms under study. Weeds are grouped by botanical family, life cycle and biological group (BG).

Binomial Name	Botanical Family	Life Cycle	BG ^†^	MOD Farm Group					OLD Farm Group					RD (%) ^‡^
				MRG	Bannò	Spitaleri	Mocciaro	Agrimor	Di Nolfo	Delizia	Minio	Antichi Granai	Cottonaro	
*Amaranthus retroflexus* L.	Amaranthaceae	annual	T	-	0.12	-	-	-	0.07	-	-	-	-	1.3
*Anagallis arvensis* L.	Primulaceae	annual	T	0.27	0.09	0.45	0.29	0.32	-	-	-	0.72	0.27	28.1
*Euphorbia falcata* L.	Euphorbiaceae	annual	T	-	-	-	-	0.04	-	0.06	-	0.12	-	0.8
*Euphorbia helioscopia* L.	Euphorbiaceae	annual	T	0.17	0.45	0.05	0.47	0.21	-	0.54	0.38	0.04	0.30	31.7
*Fallopia convolvulus* (L.) Á. Löve	Polygonaceae	annual	T	-	0.09	-	-	-	-	-	0.28	-	0.21	5.1
*Fumaria* sp.	Fumariacee	annual	T	0.06	-	0.06	-	0.08	0.24	0.06	0.10	-	0.07	4.4
*Galium aparine* L.	Rubiaceae	annual	T	-	-	-	-	-	0.24	0.06	-	-	-	2.0
*Glebionis coronaria* (L.) Cass. ex Spach	Asteraceae	annual	T	-	0.04	0.13	-	-	-	-	-	-	-	1.0
*Helminthotheca echioides* (L.) Holub	Asteraceae	annual	T	0.38	-	0.12	-	0.08	-	-	0.24	0.12	-	8.6
*Portulaca oleracea* L.	Portulacaceae	annual	T	-	0.22	-	0.24	-	-	-	-	-	-	4.0
*Sinapis arvensis* L.	Brassicaceae	annual	T	0.12	-	0.20	-	0.18	-	0.22	-	-	0.07	5.9
*Stellaria media* (L.) Vill.	Caryophyllaceae	biennial	H	-	-	-	-	-	0.45	0.06	-	-	0.07	6.7
*Veronica* sp.	Plantaginaceae	annual	T	-	-	-	-	0,09	-	-	-	-	-	0.6

^†^ T: therophytes; H: hemicryptophytes; ^‡^ Averaged over all ten farms under study.

**Table 2:** α- and β-diversity indices of weed species in the total seedbank (0–15 cm) across the 10 farms under study.

	α-Diversity			β-Diversity		
	Margalef	Shannon-Weiner	Pielou	Whittaker	Sørensen ^‡^	Steinhaus ^‡^
**MOD farm group ^†^**	**1.72 ± 0.23 A**	**1.13 ± 0.20 A**	**0.70 ± 0.15 A**	**2.6 ± 0.71 A**	76.2%	54.7%
*MRG*	1.71 b	0.99 b	0.61 b	2.6 b		
*Bannò*	1.45 b	1.02 b	0.57 b	2.2 c		
*Spitaleri*	1.68 b	1.15 b	0.64 b	2.2 c		
*Mocciaro*	1.66 b	1.03 b	0.94 a	4.3 a		
*Agrimor*	2.08 a	1.48 a	0.76 b	1.9 d		
**OLD farm group ^†^**	**1.91 ± 0.60 A**	**1.05 ± 0.38 A**	**0.68 ± 0.23 A**	**2.9 ± 0.65 A**		
*Di Nolfo*	1.51 cd	1.07 b	0.77 a	3.3 a		
*Delizia*	1.73 c	0.77 c	0.43 b	2.2 b		
*Minio*	2.05 b	1.27 ab	0.91 a	3.3 a		
*Antichi granai*	1.39 d	0.59 c	0.43 b	3.5 a		
*Cottonaro*	2.88 a	1.53 a	0.86 a	2.2 b		

Different capital letters indicate significant differences between the MOD and OLD groups at *p* ≤ 0.05 (Tukey’s HSD). Different lowercase letters indicate significant differences within groups at *p* ≤ 0.05 (Tukey’s HSD test). ^†^ Data are the mean ± standard deviation. For within groups values, the standard deviation is always 0.1. ^‡^ Similarity between the MOD and OLD groups.

**Table 3:** Mean relative abundance values and mean relative densities (RD) of weed species in the real flora across the 10 farms under study. Weeds are grouped by botanical family, life cycle and biological group (BG).

Binomial Name	Botanical Family	Life Cycle	BG ^†^	MOD Farm Group					OLD Farm Group					RD (%) ^‡^
				MRG	Bannò	Spitaleri	Mocciaro	Agrimor	Di Nolfo	Delizia	Minio	Antichi Granai	Cottonaro	
*Anagallis arvensis* L.	Primulaceae	annual	T	-	0.17	-	-	-	-	-	0.09	-	-	2.2
*Artemisia vulgaris* L.	Asteraceae	perennial	H	0.24	-	-	-	-	-	-	-	-	-	2.4
*Avena fatua* L.	Poaceae	annual	T	0.09	0.26	0.31	0.54	0.26	-	0.37	-	-	-	21.7
*Centaurea* sp.	Asteraceae	annual	T	0.18	-	0.12	-	0.15	-	0.05	0.14	0.10	-	5.9
*Convolvulus arvensis* L.	Convolvulaceae	perennial	G	-	-	-	-	-	-	-	0.13	0.26	-	4.4
*Daucus carota* L.	Apiaceae	biennal	H	0.07	-	-	-	-	0.11	0.05	0.13	0.07	-	3.2
*Diplotaxis erucoides* (L.) DC.	Brassicaceae	annual	T	-	-	-	-	-	-	-	0.03	-	-	0.1
*Euphorbia helioscopia* L.	Euphorbiaceae	annual	T	-	-	-	-	-	-	-	-	0.25	-	2.9
*Erodium cicutarium* (L.) L’Hér.	Geraniaceae	annual	T	-	-	-	-	-	0.09	0.06	-	0.11	-	1.8
*Galium aparine* L.	Rubiaceae	annual	T	-	-	-	-	-	0.54	0.19	-	-	-	8.0
*Glebionis coronaria* (L.) Cass. ex Spach	Asteraceae	annual	T	0.09	0.14	0.27	-	-	-	0.06	0.12	-	-	6.5
*Inula helenium* L.	Asteraceae	perennial	H	-	-	-	-	0.05	-	-	-	-	-	0.3
*Lolium* sp.	Poaceae	annual	T	0.07	-	-	0.31	0.05	-	0.22	-	-	-	5.8
*Papaver rhoeas* L.	Papaveraceae	annual	T	0.11	-	-	-	-	-	-	-	-	-	1.4
*Phalaris paradoxa* L.	Poaceae	annual	T	-	-	0.06	0.15	0.23	-	-	-	0.10	-	5.0
*Polygonum aviculare* L.	Polygonaceae	annual	T	-	0.24	-	-	-	-	-	-	-	-	2.6
*Sinapis arvensis* L.	Brassicaceae	annual	T	-	0.06	-	-	0.20	-	-	-	-	-	3.2
*Sonchus* sp.	Asteraceae	annual	T	0.15	0.14	-	-	-	-	-	0.12	0.07	-	4.4
*Stellaria media* (L.) Vill.	Caryophyllaceae	biennal	H	-	-	0.23	-	0.07	0.27	-	0.24	0.05	-	8.4

^†^ T: therophytes; H: hemicryptophytes; G: geophytes. ^‡^ Averaged over all ten farms under study.

**Table 4:** α- and β-diversity indices of weed species in the real flora across the 10 farms under study.

	α-Diversity			β-Diversity		
	Margalef	Shannon-Weiner	Pielou	Whittaker	Sørensen ^‡^	Steinhaus ^‡^
**MOD farm group ^†^**	**2.45 ± 0.68 A**	**1.49 ± 0.36 A**	**0.88 ± 0.04 A**	**3.8 ± 1.5 A**	64.3%	42.3%
*MRG*	3.60 a	1.95 a	0.94 a	2.4 d		
*Bannò*	2.45 b	1.65 b	0.92 a	3.2 c		
*Spitaleri*	1.92 c	1.39 b	0.87 a	3.8 b		
*Mocciaro*	2.36 b	0.96 c	0.87 a	6.3 a		
*Agrimor*	1.93 c	1.48 b	0.83 a	3.2 c		
**OLD farm group ^†^**	**1.55 ± 0.93 B**	**1.19 ± 0.76 B**	**0.65 ± 0.39 B**	**2.7 ± 1.6 A**		
*Di Nolfo*	1.34 b	0.94 c	0.68 c	4.8 a		
*Delizia*	2.20 a	1.40 b	0.72 bc	2.7 c		
*Minio*	2.06 a	1.85 a	0.89 ab	2.4 d		
*Antichi granai*	2.14 a	1.77 a	0.99 a	3.2 b		
*Cottonaro*	-	-	-	-		

Different capital letters indicate significant differences between the MOD and OLD groups at *p* ≤ 0.05 (Tukey’s HSD). Different lowercase letters indicate significant differences within the groups at *p* ≤ 0.05 (Tukey’s HSD test). ^†^ Data are the mean ± standard deviation. For within-group values, the standard deviation is always 0.1. ^‡^ Similarity between the MOD and OLD groups.

**Table 5:** Eigenvectors and eigen analysis of the first three PCs of 12 variables (6 and 7 major weeds for the soil seedbank and real flora, respectively) from PCA on the correlation matrix. Variables with the largest influence for each principal component are in bold.

Variable	Weed Communities					
	Soil Seedbank			Real Flora		
	PC1	PC2	PC3	PC1	PC2	PC3
ANGAR	**0.514**	0.070	0.081	-	-	-
AVEFA	-	-	-	**−0.504**	−0.171	−0.474
CENXX	-	-	-	0.216	**−0.545**	0.274
EUPHE	0.288	**−0.577**	−0.500	-	-	-
FALCO	−0.144	**−0.687**	0.026	-	-	-
GALAP	-	-	-	0.188	**0.618**	−0.202
GLECO	-	-	-	0.206	−0.502	**−0.594**
HELMEC	0.488	0.181	0.313	-	-	-
LOXX	-	-	-	**−0.507**	0.118	−0.278
PHALPHA	-	-	-	−0.383	−0.157	0.389
SINAR	0.311	0.308	**−0.731**	-	-	-
STELME	**−0.544**	0.292	−0.333	**0.467**	0.039	−0.279
Eigenvalue	2.346	1.491	1.038	2.505	1.693	1.178
% Variance	39.2	24.8	17.3	35.8	24.2	16.8
% Cumulative variance	39.1	64.0	81.3	35.8	60.0	76.8

**Table 6:** Geographical coordinates and agronomic management of the 5 farms belonging to the MOD group.

Farm	Geographical Coordinates	Wheat Genotype	Seeding Density (kg ha^−1^)	Tillage	Fertiliser Type and Fertilisation Time	Fertiliser Amount (kg ha^−1^)	Active Principle for Weed Chemical Control
MRG	37°35′45″ N 14°28′11″ E	Antalis	200	Hoeing (late August-early September)	Ammonium nitrate (34%) in post-emergence between February and March	120	Thifensulfuron-methyl, Tribenuron-methyl (Amedeus Top); Clodinafop-propargyl, Cloquintocet-mexyl, Pinoxaden (Traxos Pronto 60)
				Deep ploughing (−25 cm)			
				Light ploughing (−15 cm)			
Bannò	37°35′20″ N 14°27′45″ E	Anco Marzio	260	Disc ploughing	Urea (46%) in post-emergence between February and March	150	Mefenpir-diethyl, Mesosulfuron-methyl (half-dosed Atlantis); 2,4-D.
				Deep ploughing (−25 cm)			
				Light ploughing (−15 cm)			
Spitaleri	37°35′28″ N 14°28′00″ E	Iride, Simeto and Core	300	Hoeing (late August-early September)	Urea (46%) in post-emergence between February and March	250	Clodinafop-propargyl, Cloquintocet-mexyl, Pinoxaden (Traxos Pronto 60)
				Disc ploughing			
				Ploughing cultivator			
				Rolling after seeding			
Mocciaro	37°41′09″ N 14°23′53″ E	Core	240	Subsoiling (September)	Urea (46%) in post-emergence between February and March	150	Mefenpir-diethyl, Mesosulfuron-methyl (Atlantis)
				Deep ploughing (−25 cm)			
				Light ploughing (−15 cm)			
Agrimor	37°35′26″ N 14°27′01″ E	Core	230–280	Hoeing (April–May) Deep ploughing (−25 cm) on August-September Light ploughing (−15 cm) on October	Diammonium phosphate (18% N, 46% P_2_O_5_) at seeding	115–140	Clodinafop-propargyl, Cloquintocet-mexyl, Pinoxaden (Traxos Pronto 60)
					Urea (46%) in post-emergence between February and March	120	

**Table 7:** Geographical coordinates and agronomic management of the 5 farms belonging to the OLD group.

Farm	Geographical Coordinates	Wheat Genotype	Seeding Density (kg ha^−1^)	Tillage	Fertiliser Type and Fertilisation Time	Fertiliser Amount (kg ha^−1^)	Active Principle for Weed Chemical Control
Di Nolfo	37°35′22″ N 14°32′52″ E	Perciasacchi	220–230	Subsoiling	-	-	-
				Deep ploughing (−25 cm)			
				Light ploughing (−10–15 cm)			
Delizia	37°31′55.2″ N 14°12′52.7″ E	Perciasacchi	200	Disc ploughing (after wheat harvest)	-	-	-
				Subsoiling (September)			
				Deep ploughing (October)			
				Light ploughing (−10–15 cm)			
				Stale seedbed with precision seeder			
Minio	37°35′29″ N 14°30′48″ E	Perciasacchi	200–220	Hoeing (late August–early September)	-	-	-
				Deep ploughing (−25 cm)			
				Light ploughing (−10–15 cm)			
Antichi granai	37°36′08.5″ N 14°34′53.7″ E	Timilia	200	Subsoiling	Organic N (8.5%); organic C (28%)-(AMMINO-BIO) in post-emergence in April	20	-
				Deep ploughing (−25 cm)			
				Light ploughing (−10–15 cm)			
				Pre-seeding ploughing			
Cottonaro	37°35′48″ N 14°19′03″ E	Senatore Cappelli	160	Subsoiling (September)	-	-	Fluroxipir meptyl-eptyl ester, Clopiralid pure ethylammonium salt, MCPA pure potassium salt (Ariane II); Pyroxsulam, Florasulam, Cloquintocet mexyl (Floramix)
				Deep ploughing (−25 cm)			
				Light ploughing (−10 cm)			

**Table 8:** Crop sequence of the ten farms under study over the last ten years.

Farm	Crop Sequence Alternating with Wheat
**MOD farm group**	
*MRG*	vetch-clover and vetch-fava bean mix
*Bannò*	vetch and leguminous mix
*Spitaleri*	fava bean and vetch
*Mocciaro*	vetch-clover-sulla-ryegrass-oat mix
*Agrimor*	vetch
**OLD farm group**	
*Di Nolfo*	vecth-sulla-clover mix
*Delizia*	vetch-clover-sulla-ryegrass-oat mix
*Minio*	sulla, fava bean and vecth
*Antichi granai*	fallow, chickpea and lentil
*Cottonaro*	vetch-clover-sulla mix

## Data Availability

All data are available via email request to the corresponding author.
